# Embryonic Stem Cells Markers SOX2, OCT4 and Nanog Expression and Their Correlations with Epithelial-Mesenchymal Transition in Nasopharyngeal Carcinoma

**DOI:** 10.1371/journal.pone.0056324

**Published:** 2013-02-12

**Authors:** Weiren Luo, Siyi Li, Bailu Peng, Yanfen Ye, Xubin Deng, Kaitai Yao

**Affiliations:** 1 Department of Pathology, Secondary Clinical College, Guangdong Medical College, Dongguan, People's Republic of China; 2 Cancer Research Institute, Southern Medical University, Guangzhou, People's Republic of China; Health Canada, Canada

## Abstract

Expression of embryonic stem cells (ESCs) markers (SOX2, OCT4, Nanog and Nestin) is crucial for progression of various human malignancies. The purpose of this study was to investigate the expression and prognostic impact of these molecules in nasopharyngeal carcinoma (NPC) patients by immunohistochemistry and immunofluorescence. In the present study, we found that the expression levels of SOX2, OCT4 and Nanog were highly expressed in NPC compared with the non-tumorous tissues. Furthermore, these proteins correlated significantly with several clinicalpathological factors and epithelial-mesenchymal transition (EMT)-associated indicators (E-cadherin/N-cadherin and Snail). In multivariate analyses, high expression of OCT4 (*P* = 0.013) and Nanog (*P* = 0.040), but not that of SOX2, was associated with worse survival and had strongly independent prognostic effects. Of note, OCT4 and Nanog were more frequently located at the invasive front of tumors, and correlated significantly with various aggressive behaviors including T classification, N classification, M classification and clinical stage. Furthermore, patients with co-expression of OCT4 and Nanog in the invasive front had significantly worse survival (*P* = 0.005). Interestingly, at the invasive front, these molecules correlated significantly with Nestin expression in endothelial cells (*P*<0.001). These findings provide evidence that ESCs biomarkers OCT4 and Nanog serves as independent prognostic factors for NPC. Additionally, cancer cells in the invasive front of NPC acquiring ESCs-like features should be maintained by vascular niches.

## Introduction

Nasopharyngeal carcinoma (NPC) is the most frequent head and neck tumor in Guangdong, South China, which shows high incidence rate of approximately 20 to 50 cases per 100,000 people annually, with 34.01×10^5^ for male population and 11.05×10^5^ for female population, respectively. In contrast, it is rare in the Western world (less than one per 100 000 population) [1], [2]. It is likely that genetic conditions might be critical for the development of NPC, and the risk of developing NPC showed that was 9.31 times higher in the first degree relatives of patient with NPC than in that of their spouses in Guangdong province, China [3], [4]. The majority of NPC patients tend to have cervical lymph nodal metastasis when diagnosed. Though NPC patients are sensitive to radio/chemo-therapy, treatment failure remains high due to the development of local recurrence and distant metastasis [5]. To date, the molecular mechanisms related to the progression and clinical outcome of NPC have not yet been fully understood. Therefore, it is of significance to further detect valuable prognostic predictors of NPC patients.

Cancer stem cells (CSCs), defined by a small fraction of cells within the bulk tumor have the ability of self-renewal and generating new tumors, are being the hot spots in recent cancer research [6]. It has been considered that CSCs might be responsible for cancers' relapse and metastasis. These features also characterize embryonic stem cells (ESCs), thus suggesting common molecules might exist between CSCs and ESCs [7], [8]. SOX2 (SRY-related HMG-box gene 2), initially reported to be linked strongly with the inhibition of neuronal differentiation, has been shown to acts as an important transcriptional factor to maintain the self-renewal capability of ESCs [9]. OCT4 (OCT3/4), a member of the family of POU domain transcription factor and known to bind in partnership with SOX2, is also the key regulator essential for the pluripotency and self-renewal of of ESCs [10]. Nanog, a homeodomain-containing protein, maintains pluripotency of mouse ESCs by inhibiting NFκB and cooperating with Stat3 [11]. The intermediate filament protein Nestin, initially considered as a marker of neural stem cells, is found to be abundant in ESCs-derived progenitor cells that have the capability to develop into multi-lineages [12]. Of interest, accumulating evidence indicates that these transcription factors of ESCs have a strong correlation with CSCs, knockdown of these genes could decrease tumor sphere formation and inhibit tumor formation in xenograft tumor models [13]–[15]. More importantly, upregulation of these proteins was associated closely with tumor metastasis and poor prognosis in various human malignances including prostate cancer, lung adenocarcinoma, gliomas, rectal cancer, gastric carcinoma and oral squamous cell carcinoma [16]–[21]. To our knowledge, several studies have described the expression of SOX2, OCT4 and Nanog in NPC cells lines [22], [23]. However, up to date, the correlations of these molecules with clinicopathological features and patients survival still remain poorly understood.

Epithelial-mesenchymal transition (EMT) is defined by the loss of epithelial morphology and the acquisition of a mesenchymal phenotype, which is initially found to be central program in early embryonic morphogenesis [24]. In a few years, evidence has mounted for EMT as the key means through which cancer cells acquire more highly mobile potentials to migrate and metastasize to distant sites during tumor progression [25]. E-cadherin, a classical cadherin from the cadherin superfamily, is required for maintaining epithelial cell plasticity. N-cadherin, known as an important member of the cadherin family that mediates calcium-dependent adhesion, is normally expressed in mesenchymal cells. Loss of E-cadherin and increased N-cadherin expression (E/N-cadherin switch) is now defined as a major hallmark of EMT [26], [27]. Snail, one member of the zinc finger family composed of a highly conserved COOH-terminal region, induces EMT and tumor invasion by binding the E-cadherin promoter through E-box sequences. Over the past few years, accumulating data has demonstrated that EMT correlates closely with the acquisition of stem cells-like properties in cancer cells [28], [29]. For example, the Weinberg laboratory reported that the induction of EMT in immortalized human mammary epithelial cells (HMLEs) leaded to the acquisition of stem-like characteristics by using different EMT-inducers including Snail [30]. However, the connection between EMT and stem-like cells in human solid tumor tissues has not been fully described.

In the present study, we aimed to investigate the expression and prognostic impact of ESCs-associated markers SOX2, OCT4, Nanog and Nestin in our NPC cohort. In addition, in this report, the possible correlation between these ESCs proteins and EMT-related markers E-cadherin, N-cadherin and Snail was also examined.

## Materials and Methods

### Patients and Specimens

All biopsies of 122 NPC patients and 29 non-tumoral pharynx tissues were collected from the Department of Pathology, the People's Hospital of Gaozhou City, China, between 2003 and 2005. All patients did not receive preoperative radiotherapy or chemotherapy. Informed consent was approved by the local Institutional Research Ethics Committee. The clinicopathologic variables were shown as described previously [31]. Subjects comprised 92 males and 30 females, with ages ranging from 15 to 73 years (median, 47.6 years), including 89 patients with positive EBV-VCA-IgA and 33 patients with negative antibody. The clinical data of patients was reviewed according to the UICC TNM classification (2002).. There were 9 samples in stage I, 24 samples in stage II, 65 samples in stage III, and 24 samples in stage IV. The mean follow-up for overall survival was 60.1 months (ranged 8–92 months). The end date of follow-up was October 2010, with complete follow-up.

### Tissue Microarray Construction

Briefly, one core with a diameter of 1.5 mm was chosen from the selected area of each case and inserted in a recipient paraffin block using a custom-made tissue arrayer (Beecher Instruments, Silver Spring, Maryland, USA). At last, these blocks were cut into sections (4 µm thick). The whole mount sections were examined to confirm the “leading edge” on a TMA.

### Immunohistochemistry and immunofluorescence

Immunohistochemistry was performed based on the standard streptavidin-peroxidase (S-P) method (Zymed, San Francisco, CA). The experimental steps were performed as described below: After having been deparaffinized and rehydrated, the TMA sections were subjected to high pressure for antigenic retrieval for 2 minutes. The slides were incubated overnight at 4°C with primary antibodies used as follows: SOX2 (clone E-4, dilution 1∶300; Santa Cruz); OCT4 (clone C-10, dilution 1∶50; Zymed); Nanog (clone 1E6C4, dilution 1∶500; Cell Signaling Technologies; CST); Nestin (clone 10C2, dilution 1∶25; Zymed); CD31 (clone EP78, dilution 1∶100; Zymed). Finally, sections were incubated with DAB for 2 min. In every run, primary antibodies were substituted with PBS for negative controls.

For the evaluation of IHC results, the proportion of tumor cell staining was evaluated by four grades: 0, no positive tumor cells; 1, <10% positive tumor cells; 2, 10–50% positive tumor cells; 3, >50% positive tumor cells. Likewise, the scoring criteria for staining intensity were: 0, no staining; 1, weak staining; 2, modest staining; 3, strong staining. The final score was calculated by multiplying the tumor staining area by the intensity score (0, 1, 2, 3, 4, 6, 9). According to this method of assessment, staining scores ≤4 and ≥6 were regarded as tumors with low and high expression, respectively [21]. Every section was examied under ×400 magnification using the Microscope (Nikon, Japan). Two pathologists (C.X, L.W) scored all samples blindly without knowing clinical characteristics and prognosis.

According to immunofluorescence, the staining procedure was the same as IHC protocol until sections were incubated with primary antibodies overnight at 4°C. After washing in PBS, these slides were stained with Dylight594-conjugated goat anti-rabbit antibody (Jackson, dilution 1∶500) for 1 h at room temperature. Finally, slides were stained with DAPI (Sigma) for 5 minutes and mounted in Antifade Medium (P0126, Beyotime).

### Statistical Analysis

All statistical analyses were analyzed using SPSS13.0 software (SPSS Inc, Chicago, IL). The χ2 test and Fisher's exact test (when cells have expected count less than 5) were used to analyze comparisons of groups. The Kaplan–Meier method was used to estimate the overall survival rate, and differences between survival curves were estimated with the log-rank test. Multivariate survival analysis was carried out to test for independent prognosis using Cox proportional hazards regression. Correlations between variable factors were calculated by Spearman correlation coefficients. *P* values<0.05 were considered statistically significant.

## Results

### Expression and localization of SOX2, OCT4, Nanog and Nestin in NPC Tissues

**Figure 1:**
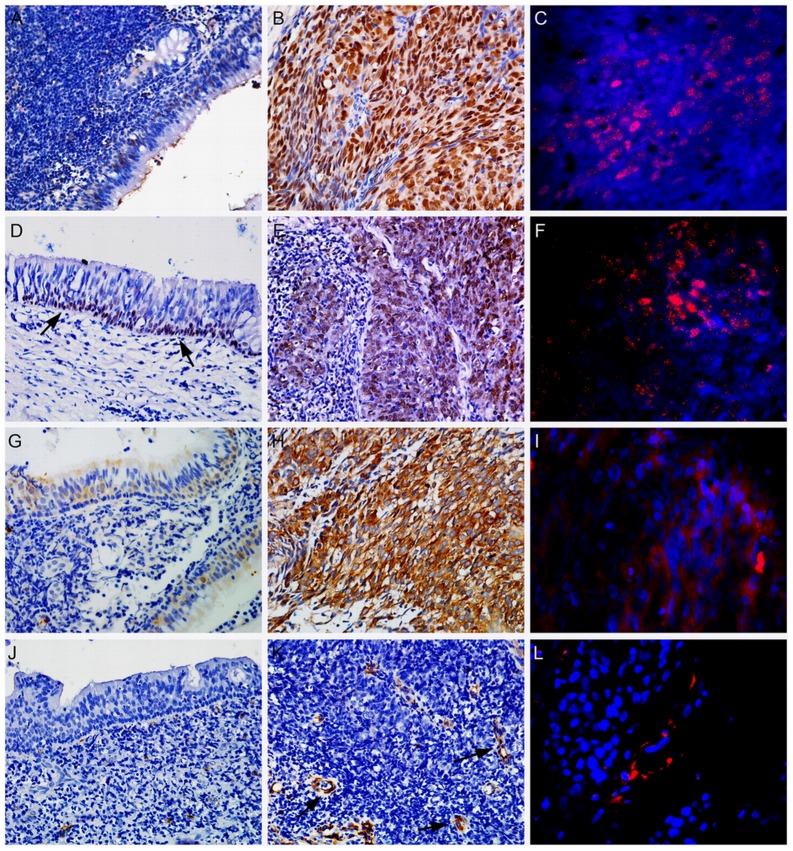
Immunohistochemical staining of embryonic stem cells (ESCs) proteins SOX2, OCT4, Nanog and Nestin in non-cancerous nasopharyngeal tissues and nasopharyngeal carcinoma (NPC). Nuclear SOX2 expression was low in non-tumoral epithelium (A), whereas it was highly expressed in NPC tissues (B). Nuclear staining of OCT4 was limited to basal cells of non-tumoral epithelium (D; arrows indicated) and markedly expressed in tumor cells (E). Low cytoplasmic expression of Nanog was observed in non-tumoral epithelium (G) and strongly displayed in tumor tissues (H). Nestin expression was completely absent in non-cancerous epithelium (J) and tumor cells (K), whereas it was strongly stained in the cytoplasm of endothelial cells in cancer tissues (K; arrows indicated). Immunofluorescent labeling of both SOX2 (C) and OCT4 (C) showed nuclear staining (red), Nanog (I) and Nestin (L) showed cytoplasmic localization (red) of tumor cells and endothelial cells, respectively, DAPI (blue).All images, ×400.

**Figure 2:**
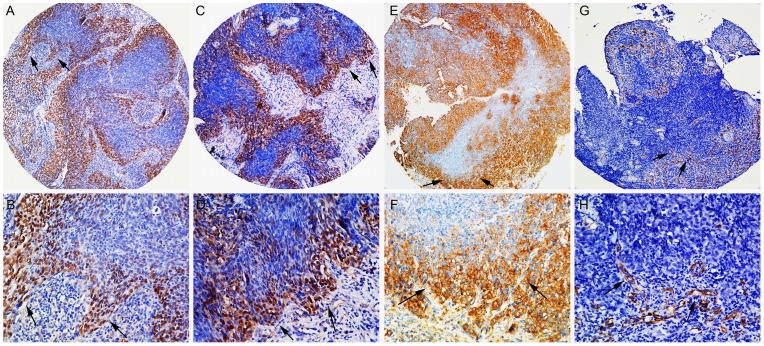
Immunohistochemical expression levels of SOX2, OCT4, Nanog and Nestin in the invasive front of NPC (arrows indicated). Strong staining of nuclear SOX2 (A, B) was mostly found at the tumor invasive front. High staining of nuclear OCT4 (C, D) was observed in the invasive front of tumors. Cytoplasmic Nanog expression (E, F) was particularly evident at the invasive edge of tumors. **Of note**, these cells often exhibited a fibroblast-like, spindle-shaped phenotype. On the other hand, Nestin expression in blood vessels (G, H) were distributed predominantly at the invasive front of tumors. (A, C, E, G×100; B, D, F, H×400, respectively).

The immunohistochemical results for ESCs-related markers are summarized in Table 1–2. No positive or low staining of SOX2 was detected in non-cancerous epithelium (Fig. 1A), only 5 cases (17.2%) highly exhibited nuclear staining. In contrast, of the 122 tumors, 68 samples (55.7%, *P* = 0.000) showed high nuclear expression of SOX2 (Fig. 1B). Interestingly, immunohistochemical analysis of 122 NPCs showed an invasion front-specific overexpression of nuclear SOX2 in 37 samples (Fig. 2A, B) compared with the tumor center-specific overexpression of that in 20 samples (*P* = 0.010).

**Table 1:** Associations between the clinicopathologic factors and SOX2, OCT4 and Nanog expression in 122 NPC patients.

Variables	N	SOX2 expression (N, %)			OCT4 expression (N, %)			Nanog expression (N, %)		
		Low	High	*P*	Low	High	*P*	Low	High	*P*
Gender										
Male	92	40 (43.5)	52 (56.5)	0. 760	59 (64.1)	33 (35.9)	0.801	41 (44.6)	51 (55.4)	0.279
Female	30	14 (46.7)	16 (53.3)		20 (66.7)	10 (33.3)		10 (33.3)	20 (66.7)	
Age (y)										
<48	63	28 (44.4)	35 (55.6)	0.967	45 (71.4)	18 (28.6)	0.111	31 (49.2)	32 (50.8)	0.376
≥48	59	26 (44.1)	33 (55.9)		34 (57.6)	25 (42.4)		20 (40.8)	29 (59.2)	
Histologic type										
NKC	19	8 (42.1)	11 (57.9)	0.837	13 (68.4)	6 (31.6)	0.716	13 (68.4)	6 (31.6)	0.010
UC	103	46 (44.7)	57 (55.3)		66 (64.1)	37 (35.9)		38 (36.9)	65 (63.1)	
T classification										
T1–T2	58	31 (53.4)	27 (46.6)	0.214	44 (75.9)	14 (24.1)	0.014	33 (56.9)	25 (43.1)	0.001
T3–T4	64	27 (42.2)	37 (57.8)		35 (54.7)	29 (45.3)		18 (28.1)	46 (71.9)	
N classification										
N0–N1	70	41 (58.6)	29 (41.4)	0.000	53 (75.7)	17 (24.3)	0.003	40 (57.1)	30 (42.9)	0.000
N2–N3	52	13 (25.0)	39 (75.0)		26 (50.0)	26 (50.0)		11 (21.2)	41 (78.8)	
M classification										
M0	107	51 (47.7)	56 (52.3)	0.043	73 (68.2)	34 (31.8)	0.032	46 (43.0)	61 (57.0)	0.478
M1	15	3 (20.0)	12 (80.0)		6 (40.0)	9 (60.0)		5 (33.3)	10 (66.7)	
Tumor stage										
I–II	33	24 (72.7)	9 (27.3)	0.000	31 (93.9)	2 (6.1)	0.000	25 (75.8)	8 (24.2)	0.000
III–IV	89	30 (33.7)	59 (66.3)		47 (52.8)	42 (47.2)		26 (29.2)	63 (70.8)	
E-cadherin staining										
Low group	95	38 (40.0)	57 (60.0)	0.075	57 (60.0)	38 (40.0)	0.038	31 (32.6)	64 (67.4)	0.000
High group	27	16 (59.3)	11 (40.7)		22 (81.5)	5 (18.5)		20 (74.1)	7 (25.9)	
N-cadherin staining										
Low group	67	40 (59.7)	27 (40.3)	0.000	52 (77.6)	15 (22.4)	0.001	39 (58.2)	28 (41.8)	0.000
High group	55	14 (25.5)	41 (74.5)		27 (49.1)	28 (50.9)		12 (21.8)	43 (78.2)	
Snail staining										
Low group	62	35 (56.5)	27 (43.5)	0.006	48 (77.4)	14 (22.6)	0.003	32(51.6)	33(48.4)	0.026
High group	60	19 (31.7)	41 (68.3)		31 (51.7)	29 (48.3)		19(31.7)	41(68.3)	

Abbreviations: NKC, differentiated nonkeratinizing carcinoma; UC, undifferentiatied carcinoma; T, tumor size; N, lymph node; M, distant metastasis.

**Table 2:** Associations between the clinicopathologic factors and SOX2, OCT4 and Nanog at the tumor invasive front in 122 NPCs.

Variables	N	SOX2 expression (N, %)			OCT4 expression (N, %)			Nanog expression (N, %)		
		Low	High	*P*	Low	High	*P*	Low	High	*P*
Gender										
Male	92	63 (68.5)	29 (31.5)	0.615	69 (75.0)	23 (25.0)	0.856	60 (65.2)	32 (34.8)	0.243
Female	30	22 (73.3)	8 (26.6)		22 (73.3)	8 (26.7)		16 (53.3)	14 (46.7)	
Age (y)										
<48	63	45 (71.4)	18 (28.6)	0.663	51 (81.0)	12 (19.0)	0.095	44 (69.8)	19 (30.2)	0.076
≥48	59	40 (67.8)	19 (32.2)		40 (67.8)	19 (32.2)		32 (54.2)	27 (45.8)	
Histologic type										
NKC	19	10 (52.6)	9 (47.4)	0.079	16 (84.2)	3 (15.8)	0.294	13 (68.4)	6 (31.6)	0.549
UC	103	75 (72.8)	28 (27.2)		75 (72.8)	28 (27.2)		63 (61.2)	40 (38.8)	
T classification										
T1–T2	58	45 (77.6)	13 (22.4)	0.203	51 (87.9)	7 (12.1)	0.001	45 (56.9)	33 (43.1)	0.001
T3–T4	64	50 (67.6)	24 (32.4)		40 (62.5)	24 (37.5)		18 (28.1)	46 (71.9)	
N classification										
N0–N1	70	54 (77.1)	16 (22.9)	0.037	56 (80.0)	14 (20.0)	0.111	51 (72.9)	19 (27.1)	0.005
N2–N3	52	31 (59.6)	21 (40.4)		35 (67.3)	17 (32.7)		25 (48.1)	27 (51.9)	
M classification										
M0	107	76 (71.0)	31 (29.0)	0.384	87 (81.3)	20 (18.7)	0.000	71 (66.4)	36 (33.6)	0.013
M1	15	9 (60.0)	6 (40.0)		4 (26.7)	11 (73.3)		5 (33.3)	10 (66.7)	
Tumor stage										
I–II	33	31 (93.9)	2 (6.1)	0.000	29 (87.9)	4 (12.1)	0.040	28 (84.8)	5 (15.2)	0.002
III–IV	89	54 (60.7)	35 (39.3)		62 (69.7)	27 (30.3)		48 (53.9)	41 (46.1)	
Nestin staining #										
Low group	90	75 (83.3)	15 (16.7)	0.000	79 (87.8)	11 (12.2)	0.000	64 (71.1)	26 (28.9)	0.001
High group	32	10 (31.3)	22 (68.7)		12 (37.5)	20 (62.5)		12 (37.5)	20 (62.5)	

# Nestin expressed in endocelluar cells in the invasive front;

Abbreviations: NKC, differentiated nonkeratinizing carcinoma; UC, undifferentiatied carcinoma; T, tumor size; N, lymph node; M, distant metastasis.

Nuclear localization of OCT4 was highly expressed in 35.2% (43/122) of tumor cases (Fig. 1E). In contrast, nuclear OCT4 expression was negative in non-cancerous epithelial tissues, or mainly expressed in the basal cell layers (Fig. 1D), only 4 cases (13.8%) exhibited a positive reaction. It is of note that nuclear OCT4 was located predominantly at the tumor invasive edge (Fig. 2C, D). Thirty-one of the total cases showed nuclear OCT4 predominantly occurred at the tumor invasive front (Fig. 2C, D), whereas only 15 cases showed high expression of nuclear OCT4 in the tumor center (*P* = 0.009).

Cytoplasmic expression of Nanog was weakly detected in 72.4% (21/29) of non-cancerous cases (Fig. 1G), with high expression in only 8 samples. On the contrary, the majority of cases (63/122, 51.6%) were strong for cytoplasmic Nanog expression (Fig. 1H), and 8 cases (6.6%) exhibiting nuclear staining. Of note, in 122 tumor samples, 46 revealed an invasive edge-prefer overexpression of Nanog (Fig. 2E, F) compared with 21 cases with distinct-overexpression in the tumor center (*P* = 0.000).

Cytoplasmic Nestin was negatively detectable in non-cancerous cases (Fig. 1J), only 2 samples were faintly detectable. Similarly, Nestin expression was absent in tumor cells (Fig. 1K) except for 2 cases. In contrast, endothelial cells (confirmed by CD31 antibody) showed highly positive immunoreactivity for cytoplasmic Nestin (39.3%, 48/122). In 48 cancer tissues, 32 cases (66.7%) showed Nestin positive-endothelium located frequently in the invasive front (Fig. 2G, H), and 6 csses in intra-tumor and 10 cases in both of them.

Immunofluorescence labeling was performed to detect the definitive localization of these molecules. Consistent with immunohistochemical staining, SOX2 and OCT4 were localized in the nucleus of tumor cells (Fig. 1C, F), Nanog was localized in the cytoplasm of cancer cells and Nestin was found to be present in the cytoplasm of endothelial cells, respectively (Fig. 1I, L).

### Associations between ESCs Proteins SOX2, OCT4, Nanog with EMT Related Biomarkers

Data on EMT-related markers E-cadherin, N-cadherin and Snail were obtained from recent studies for comparisons [31]–[33]. As shown in Table 1, high SOX2 expression displayed a significant association with high expression of N-cadherin (*P* = 0.000), whereas not with E-cadherin expression (*P* = 0.075). There was a significant association between high OCT4 expression and low E-cadherin expression (*P* = 0.038), also for high N-cadherin expression (*P* = 0.001). Nanog expression correlated inversely with low E-cadherin expression (*P* = 0.000) and positively with high N-cadherin expression (*P* = 0.000). High Snail expression correlated closely with increased expression of SOX2 (*P* = 0.006), OCT4 (*P* = 0.003) and Nanog (*P* = 0.026).

### Correlations between SOX2, OCT4, Nanog and Nestin

In primary tumors, high expression of SOX2 was significantly associated with increased OCT4 expression (Spearman correlation coefficient, 0.243; *P* = 0.007), whereas there was no significant correlation between the expression of SOX2 and Nanog (Spearman correlation coefficient, 0.148; *P* = 0.104). In turn, a significantly positive association was observed between OCT4 and Nanog expression (Spearman correlation coefficient, 0.382; *P* = 0.000).

In the tumor invasive front (the leading edge), a significant association was identified between the expression of SOX2 and OCT4 (Spearman correlation coefficient, 0.188; *P* = 0.038), as well as SOX2 and Nanog expression (Spearman correlation coefficient, 0.223; *P* = 0.014). Similarly, there was a significant correlation between high expression of OCT4 and Nanog (Spearman correlation coefficient, 0.401; *P* = 0.000).

It is of note that Nestin expression in endothelial cells correlated positively with the expression of SOX2 (*P* = 0.000), OCT4 (*P* = 0.000) and Nanog (*P* = 0.001) in the invasive front of tumors, as listed in Table 2. More interestingly, in several samples, we found that cancer cells with high expression of ESCs-related marker SOX2, OCT4 and Nanog, particularly SOX2, were surrounded by Nestin positive-endothelium.

### Associations of the Clinicopathological Features with SOX2, OCT4 and Nanog Expression

As summarized in Table 1, high nuclear expression of SOX2 was significantly associated with several clinicopathological features including N classification (*P* = 0.000), M classification (*P* = 0.043) and tumor stage (*P* = 0.000). Nuclear OCT4 expression was found significantly more often among tumors with T classification (*P* = 0.014), N classification (*P* = 0.003), M classification (*P* = 0.032) and tumor stage (*P* = 0.000). High cytoplasmic Nanog displayed significantly positive associations with histologic subtype (*P* = 0.010), T classification (*P* = 0.001), N classification (*P* = 0.000) and tumor stage (*P* = 0.000).

On the other side, in the tumor invasive front, SOX2 expression correlated significantly with N classification (*P* = 0.037) and tumor stage (*P* = 0.000). OCT4 expression in the invasive front was significantly associated with T classification (*P* = 0.001), M classification (*P* = 0.000) and tumor stage (*P* = 0.040; Table 2). Nanog expression in the invasive margin correlated significantly with T classification (*P* = 0.001), N classification (*P* = 0.005), M classification (*P* = 0.013) and tumor stage (*P* = 0.002; Table 2).

### Correlations between SOX2, OCT4 and Nanog Expression and Patients Survival

**Figure 3:**
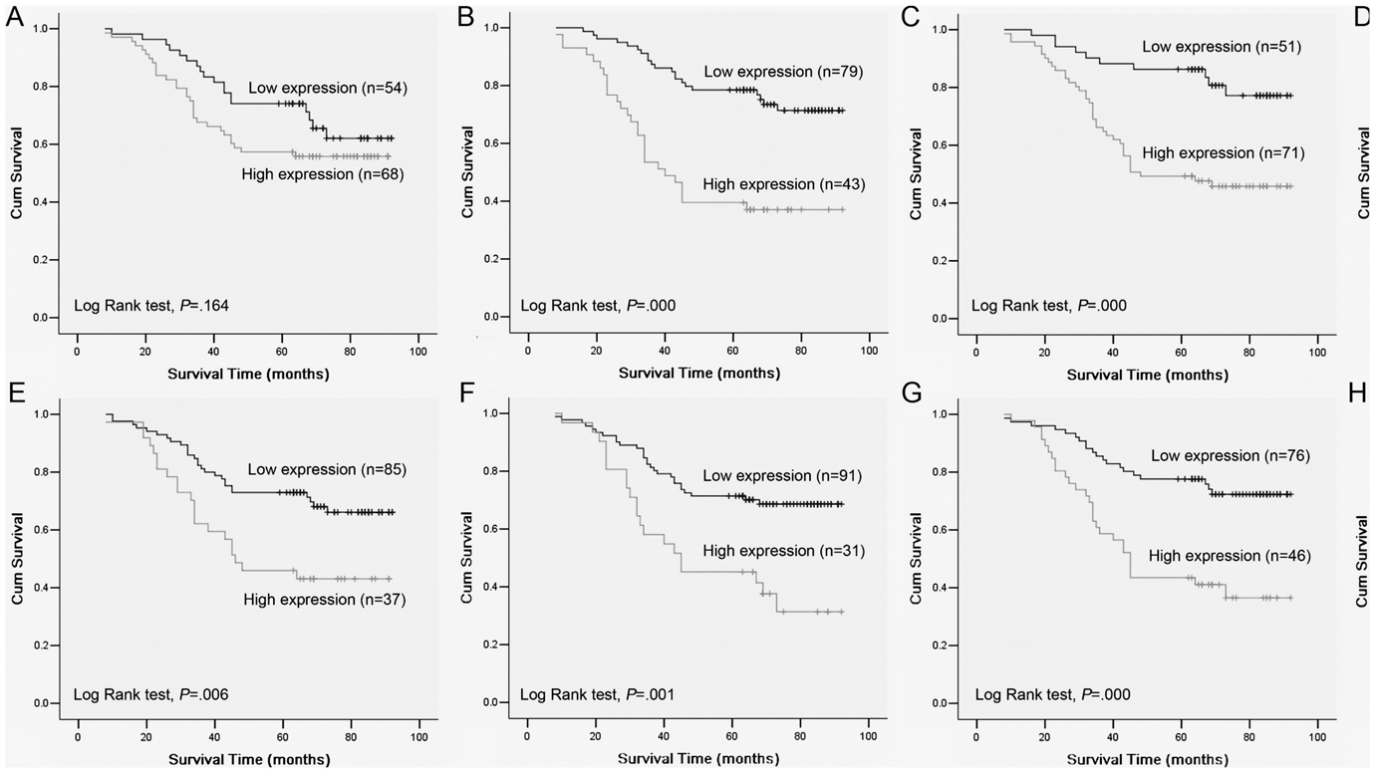
Influence of SOX2, OCT4 and Nanog expression on overall survival of NPC patients. There was no significant difference in the overall survival between low and high nuclear SOX2 expression(A). Patients showed worse overall survival with high nuclear OCT4 (B), cytoplasmic Nanog (C) and coexpression of OCT4 and Nanog (D) in tumors. Patients with high expression of nuclear SOX2 (E), nuclear OCT4 (F), cytoplasmic Nanog (G) and coexpression of OCT4 and Nanog (H) in the invasive front of tumors showed worse overall survival. *P*-values were calculated by log-rank test.

Kaplan-Meier analysis and the log-rank test were used to detect the prognostic impacts of SOX2, OCT4 and Nanog expression on patient survival. In univariate analysis, high expression of OCT4 (*P* = 0.000, Fig. 3B) and Nanog (*P* = 0.000, Fig. 3C) correlated significantly with worse overall survival of NPC patients (Table S1). However, no significant correlation was observed between SOX2 expression and overall survival of patients (*P* = 0.164, Fig. 3A). Subsequently, Cox models were performed to determine the independent factors for NPC patients. As summarized in Table S1, OCT4 expression (*P* = 0.013) and Nanog expression (*P* = 0.040) might be independent prognostic factors, as well as M classification (*P* = 0.000). To further clarify the prognostic impacts of OCT4 and Nanog expression, a final concomitant model was studied. Patients in the high coexpression of OCT4 and Nanog group had significantly reduced survival compared with those with pathologic staining of none or only one of the markers, with 36.1% 5-year survival (95% CI, 0.238–0.484) compared with 76.7% 5-year survival (95% CI, 0.678–0.856) (*P* = 0.000, Fig. 3D).

We also investigated the prognostic value of SOX2, OCT4 and Nanog expression in the invasive front on patients' survival. The results showed that, in the tumor invasive front, patients with high SOX2 staining had a worse prognosis than those with low SOX2 expression (*P* = 0.006, Fig. 3E). Besides this, high expression levels of OCT4 (*P* = 0.001, Fig. 3F) and Nanog (*P* = 0.000, Fig. 3G) in the invasive front of tumors were also found to be associated significantly with poor overall survival, respectively. Furthermore, the cumulative 5-year survival was only 40.9% (95% CI, 0.203–0.615) in the high coexpression group compared with 70.0% (95% CI, 0.610–0.790) in other remain group at the invasive margin (*P* = 0.005, Fig. 3H).

## Discussion

We here showed that increased expression of OCT4 and Nanog was significantly associated with aggressive behaviors of NPC including T classification, M classification and tumor stage. Furthermore, these two proteins were shown to be independent prognostic factors. Notably, both OCT4 and Nanog expression were predominantly observed in tumor cells at the invasive front, and correlated strongly with Nestin expression. As novel findings, OCT4 and Nanog expression might serve as valuable predictors of NPC patients.

Embryonic stem cells (ESCs) are defined as cells that have their ability to self-renew and to differentiate into a variety of adult tissues and cell types. It is generally considered that SOX2, OCT4 and Nanog are key transcription regulators that maintain the pluripotency and self-renewal properties of ESCs [9]–[11]. Growing data demonstrates that stable expression of SOX2, OCT4 and Nanog could promote tumor cell growth, anti-apoptosis and metastasis *in vitro* and *in vivo*, therefore play an important role in carcinogenesis [34]–[36]. Of importance, these ESCs-associated proteins were highly expressed in various cancers and contributed to tumor aggressiveness and poor outcome [13]–[15]. However, little is known about the expression levels of these molecules and their correlations with clinical significance in NPC patients. Compared with non-tumoral epithelium, we observed that the expression levels of SOX2, OCT4 and Nanog were highly increased in NPC tissues, respectively, suggesting that these molecules might be involved in the pathogenesis of NPC. Furthermore, our results also revealed that the high expression of SOX2, OCT4 and Nanog was closely associated with tumor aggressive behaviors of NPC patients. For example, both OCT4 and Nanog expression correlated significantly with T classification, N classification and clinical stage. Furthermore, patients with coexpression of OCT4 and Nanog had significantly worse overall survival. Similar to our observations, coexpression of Oct4 and Nanog was found to link significantly with tumor aggressiveness and poor prognosis of several malignances including lung cancer, oral cancer and hepatocellular carcinoma [16], [20], [37]. In fact, a direct link was investigated between OCT4 and Nanog, and they jointly controlled a cascade of pathways to govern the pluripotency and self-renewal characteristics of ESCs [38]. In the present study, a significantly positive relationship between high expression of OCT4 and Nanog was also found. Based on these findings, we suggest that there might be a positive involvement of OCT4/Nanog signaling in tumor invasion and progression of NPC. However, functional impacts of coexpression of OCT4 and Nanog in NPC need to be further examined. Additionally, it is well known that EB-virus infection is strongly associated with NPC carcinogenesis. Therefore, it is of importance to further detect the potential correlation between EBV infection and ESCs-associated biomarkers.

The mesenchymal phenotypic changes by increased motility and invasiveness of epithelial tumor cells are known as the epithelial-mesenchymal transition (EMT) [24], [25], [28]. As a feature of aggressive tumors, EMT is characterized by a switch from E-cadherin to N-cadherin expression, which has been found to correlate with tumor progression and metastasis [26], [27]. Our previous findings also showed that aberrant E/N-cadherin expression contributed to tumor progression and poor outcome of NPC [31], [32]. Of interest, we here showed that overexpression of SOX2, OCT4 and Nanog was significantly associated with high expression of N-cadherin, but adversely with low E-cadherin expression (except for SOX2). Additionally, overexpression of these proteins correlated strongly with the expression of Snail, a central transcription factor as E-cadherin repressor. In line with our observations, several studies *in intro* reported that overexpression of SOX2, OCT4, and Nanog, individually or simultaneously, leaded to the induction of EMT [16], [39], [40]. Particularly, we found that distributions of SOX2, OCT4 and Nanog staining were more frequently located in the invasive front of tumors, and these cells often exhibited a fibroblast-like, spindle-shaped phenotype. Recently, we have demonstrated that these spindle cells in the invasive tumor front correlated strongly with EMT in NPC tissues [41]. Taken together, our findings strongly indicate that these stem cells-like cancer cells might strongly resemble cells that have undergone an EMT. As expected, high expression of these proteins in the invasive front correlated significantly with a majority of tumor aggressive behaviors in NPC, such as tumor infiltration, lymph node metastasis and distant metastasis. To the best of our knowledge, no reports have previously described the prognostic impacts of these ESCs proteins in the invasive front of tumors. Collectively, we speculate that tumor cells with stem-like properties in the invasive tumor front of NPC are capable of generating tumor invasion and metastases.

The niche concept was firstly defined as “stem cell niche”, which is composed of diverse stromal cells including mesenchymal and immune cells, and regulates self-renewal, proliferation, and apoptosis resistance of stem cells [42], [43]. Like normal stem cells, it is currently thought that CSCs also rely on a “CSC niche”, to maintain their exclusive abilities to self-renew and grow more differentiated cells [44]–[46]. Of note, vascular niche has recently proved to be responsible for the induction of CSCs properties [47]. For example, Calabrese et al. demonstrated that brain tumor CSCs might live in a “vascular niche” that stimulates their self-renewal. Disrupting this niche impaired CSC self-renewal and significantly inhibited tumor growth [48]. Nestin, is an intermediate filament protein known as a stem/progenitor cell marker, which is normally expressed in undifferentiated central nervous system (CNS) cells, but also in endothelial cells [49]. More recently, He H et al. reported that Nestin-positive blood vessels were crucial for maintaining the structure of the glioma stem cell niche [50]. Interestingly, in the present study, Nestin expression was predominately found in endothelial cells at the invasive front of NPC. Furthermore, tumor cells exhibiting CSCs-like features correlated significantly with Nestin staining in the invasive front. Based on these findings, we postulate that vascular endothelial cells expressing Nestin might represent the CSCs niche in NPC. Surely, functional investigations are warranted to further elucidate this hypothesis.

In summary, our findings show first that OCT4 and Nanog expression might be independent prognostic predictors for patients with NPC. We also postulate that tumor cells with stem cell-like features in the invasive front could generate metastasic capability, and these stem-like features might be maintained by endothelial niches. These findings should be responsible for the clinical behaviors of NPC and could be valuable therapeutic targets.

## Supporting Information

Table S1 Univariate and multivariate survival analysis according to clinicopathologic factors, SOX2, OCT4 and Nanog in 122 NPCs.
(DOC)
