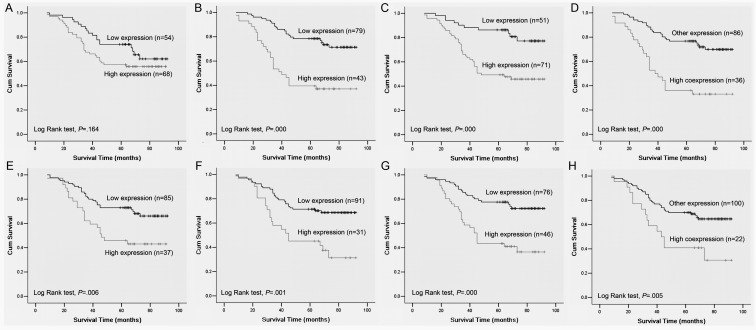# Correction: Embryonic Stem Cells Markers SOX2, OCT4 and Nanog Expression and Their Correlations with Epithelial-Mesenchymal Transition in Nasopharyngeal Carcinoma

**DOI:** 10.1371/annotation/d643b78d-fed0-4c23-a795-3da282c15a6f

**Published:** 2013-10-11

**Authors:** Weiren Luo, Siyi Li, Bailu Peng, Yanfen Ye, Xubin Deng, Kaitai Yao

An error in Figure 3 was introduced during the preparation of this article for publication. Figure 3 should include two additional graphs labeled D and H. Please see the corrected Figure 3 here: 

**Figure:**