# Supplementary material for: Embryonic Stem Cells Markers SOX2, OCT4 and Nanog Expression and Their Correlations with Epithelial-Mesenchymal Transition in Nasopharyngeal Carcinoma
Source: PLoS One. 2013 Feb 12;8(2):e56324. doi: 10.1371/journal.pone.0056324 (PMC3570418; doi:10.1371/journal.pone.0056324)
Supplement: Table S1 — Univariate and multivariate survival analysis according to clinicopathologic factors, SOX2, OCT4 and Nanog in 122 NPCs. (DOC) [file pone.0056324.s001.doc]

| **Table S1** Univariate and multivariate survival analysis according to clinicopathologic factors, SOX2, OCT4 and Nanog in 122 NPCs | | | | | | | |
| --- | --- | --- | --- | --- | --- | --- | --- |
| **Parameters** | **N** | **Univariate Analysis** | |  | **Multivariate Analysis** | | |
|  |  | **Overall Survival (m)** | ***P*** |  | **HR** | **95% CI** | ***P*** |
| Gender |  |  |  |  |  |  |  |
| Male | 92 | 63.979-75.987 | 0.577 |  | 0.989 | 0.509-1.921 | 0.973 |
| Female | 30 | 53.910-73.662 |  |  |  |  |  |
| Age (y) |  |  |  |  |  |  |  |
| ＜48 | 63 | 62.277-76.273 | 0.965 |  | 0.788 | 0.423-1.468 | 0.453 |
| ≥48 | 59 | 60.652-76.128 |  |  |  |  |  |
| Histologic type |  |  |  |  |  |  |  |
| NKC | 19 | 60.357-83.973 | 0.722 |  | 0.747 | 0.308-1.815 | 0.747 |
| UC | 103 | 62.685-74.278 |  |  |  |  |  |
| T classification |  |  |  |  |  |  |  |
| T1-T2 | 58 | 67.199-81.021 | 0.110 |  | 0.722 | 0.328-1.588 | 0.418 |
| T3-T4 | 64 | 56.922-72.186 |  |  |  |  |  |
| N classification |  |  |  |  |  |  |  |
| N0-N1 | 70 | 68.092-80.850 | 0.026 |  | 0.685 | 0.314-1.497 | 0.343 |
| N2-N3 | 52 | 53.536-70.411 |  |  |  |  |  |
| M classification |  |  |  |  |  |  |  |
| M0 | 107 | 68.630-78.734 | 0.000 |  | 4.453 | 2.105-9.423 | 0.000 |
| M1 | 15 | 21.953-51.247 |  |  |  |  |  |
| Tumor stage |  |  |  |  |  |  |  |
| Ⅰ-Ⅱ | 33 | 79.729-90.258 | 0.002 |  | 2.223 | 0.627-7.878 | 0.216 |
| Ⅲ-Ⅳ | 89 | 56.796-69.815 |  |  |  |  |  |
| SOX2 expression |  |  |  |  |  |  |  |
| Low group | 54 | 67.110-81.106 | 0.164 |  | 1.072 | 0.556-2.064 | 0.836 |
| High group | 68 | 57.079-71.790 |  |  |  |  |  |
| OCT4 expression |  |  |  |  |  |  |  |
| Low group | 79 | 72.667-83.351 | 0.000 |  | 2.266 | 1.187-4.328 | 0.013 |
| High group | 43 | 43.553-62.413 |  |  |  |  |  |
| Nanog expression |  |  |  |  |  |  |  |
| Low group | 51 | 75.188-87.536 | 0.000 |  | 2.356 | 1.041-5.332 | 0.040 |
| High group | 71 | 53.115-67.488 |  |  |  |  |  |
| Abbreviations: NKC, differentiated nonkeratinizing carcinoma; UC, undifferentiatied carcinoma; T, tumor size; N, lymph node; M, distant metastasis; HR, hazard ratio; 95%CI, 95% confidence interval. | | | | | | | |
